# Supplementary material for: Whole-Genome Sequencing of Human Enteroviruses from Clinical Samples by Nanopore Direct RNA Sequencing
Source: Viruses. 2020 Jul 31;12(8):841. doi: 10.3390/v12080841 (PMC7472277; doi:10.3390/v12080841)
Supplement: Supplementary file 1 [file viruses-12-00841-s001.pdf]

viruses

MDPI

Supplementary Table 1. Summary of laboratory experiments.

Sample	Clinical information				Sequencing approach	Run ID	Starting material, RNA extraction and processing methods	Amount of nucleic acids used for NGS	
	C_t value real-time PCR	Symptoms	Age, Gender	EV genotype				Library preparation input	Loaded on flowcell
**E590**	23.0	Fever, rash, diarrhea	20 months old, male	EV-A6, E18 (Illumina MiSeq)	DRS	E590-DRS	• Chloroform/bead pre-treatment<br>• easyMAG extraction (input volume 1000 µL, elution volume 25 µL)	450 ng RNA (Qubit RNA HS kit)	136 ng RNA (Qubit RNA HS kit)
					Illumina MiSeq	E590-MiSeq	• Routine diagnostic pre-treatment<br>• TRIzol extraction<br>• ds cDNA synthesis using 3 primers: EV-3UTR1_A6_rc, 2588R_A6, 5672R_A6	0.924 ng cDNA (Qubit DNA HS kit)	23 ng cDNA
**E372**	18.8	Fever	1 month old, male	E30 (Sanger)	DRS	E372-DRS	• Chloroform/bead pre-treatment<br>• TRIzol extraction	514 ng RNA (Qubit RNA HS kit)	ca. 40 ng (Qubit dsDNA HS kit)
					Illumina MiSeq	E372-MiSeqR6 and E372-MiSeqOdT	• Routine diagnostic pre-treatment<br>• TRIzol extraction<br>• ds cDNA synthesis: two reactions with either Oligo dT (OdT) or random hexamer (R6) primers	cDNA concentration too low, out of range (Qubit DNA HS kit)	concentration too low, out of range
**E026**	22.5	Fever, diarrhea	24 years old, male	E25 (Sanger)	DRS	E026-DRS	• Chloroform/bead pre-treatment<br>• TRIzol extraction	361 ng (Qubit RNA HS kit)	896 ng (Qubit dsDNA BR kit)
					Illumina MiSeq	E026-MiSeqR6 and E026-MiSeqOdT	• Routine diagnostic pre-treatment<br>• TRIzol extraction<br>• ds cDNA synthesis: two reactions with either Oligo dT (OdT) or random hexamer (R6) primers	cDNA concentration at limit of detection (Qubit DNA HS kit)	E026-MiSeqR6: ca. 16 ng E026-MiSeqOdT: ca. 13 ng

Supplementary Table 2. Summary of nanopore sequencing data.

Sample	Run ID	Raw data (all basecalled reads)				Passed basecalling (min. qscore 7)	
		Run duration (h)	Total reads	Number of bases	Mean read length (range)	Total read number (%)	Mean read length (range)
**E590**	E590-DRS	7	137,834	15,262,143	110.7 (1–123,377)	9,213	1,127.7 (1–7,112)
**E372**	E372-DRS	12	56,000	7,076,193	126.4 (1–24,441)	31,636	144.4 (2–4,276)
**E026**	E026-DRS	48	1,571,837	1,576,786,907	1,003.1 (1–121,608)	1,469,541	1,035.3 (1–7,101)

Supplementary Table 3. Summary of Illumina sequencing data.

Sample	Run ID	Raw data			After quality filtering, adaptor removal	
		Total reads	Number of bases	Mean read length (range)	Total read number (%)	Mean read length (range)
**E590**	E590-MiSeq	1,510,102	227,487,518	150.6 (59–151)	1,508,378 (99.89%)	125 (10-151)
**E372**	E372-MiSeqR6	3,658,398	478,895,997	130.9 (35–151)	2,086,914 (57.04%)	116.8 (10-151)
	E372-MiSeqOdT	1,917,874	269,295,604	140.4 (35–151)	819,992 (42.76%)	128.9 (10-151)
**E026**	E026-MiSeqR6	14,895,978	1,819,098,189	122.1 (35–151)	12,089,696 (81.16%)	115.9 (10-151)
	E026-MiSeqOdT	7,425,482	956,412,055	128.8 (35–151)	5,786,922 (77.93%)	123.2 (10-151)
